# Technology for the Quantitative Identification of Dairy Products Based on Raman Spectroscopy, Chemometrics, and Machine Learning

**DOI:** 10.3390/molecules30020239

**Published:** 2025-01-09

**Authors:** Zheng-Yong Zhang, Jian-Sheng Su, Huan-Ming Xiong

**Affiliations:** 1 School of Management Science and Engineering, Nanjing University of Finance and Economics, Nanjing 210023, China; zyzhang@nufe.edu.cn (Z.-Y.Z.); 1120230452@stu.nufe.edu.cn (J.-S.S.); 2 Department of Chemistry, Shanghai Key Laboratory of Molecular Catalysis and Innovative Materials, Fudan University, Shanghai 200438, China

**Keywords:** dairy products, Raman spectroscopy, quantitative identification, chemometrics, machine learning

## Abstract

The technologies used for the characterization and quantitative analysis of dairy products based on Raman spectroscopy have developed rapidly in recent years. At the level of spectral data, there are not only traditional Raman spectra but also two-dimensional correlation spectra, which can provide rich compositional and characteristic information about the samples. In terms of spectral preprocessing, there are various methods, such as normalization, wavelet denoising, and feature extraction. A combination of these methods with appropriate quantitative techniques is beneficial to reveal the differences between samples or improve predictive performance. Quantitative evaluation can be divided into similarity measurement methods and machine learning algorithms. When evaluating small batch samples, similarity measurements can provide quantitative discrimination results. When the sample data are sufficient and matched with Raman spectroscopy parameters, machine learning algorithms suitable for intelligent discrimination can be trained and optimized. Finally, with the rise of deep learning algorithms and fusion strategies, some challenges in this field are proposed.

## 1. Introduction

Dairy products, which are rich in various nutrients, are important sources of food for the general public. It is estimated that the global market size of dairy products is expected to reach over 900 billion US dollars by 2024. However, quality and safety issues related to dairy products remain important obstacles to their industrial development [1,2]. Moore et al. reviewed the food fraud from 1980 to 2010 and revealed that reports of adulteration in dairy products were second only to olive oil [3]. During this period, the issue of nonprotein nitrogen adulterants such as melamine and dicyandiamide in dairy products has attracted widespread attention [4,5,6,7]. One of the more representative incidents was the 2008 melamine dairy product contamination incident [8]. Zhu et al. analyzed the content of melamine and its derivatives (cyanuric acid, ammonine, and ammonide) in American dairy products and found that the median concentration of melamine and its derivatives in infant formula collected in 2008 (9.4 ng/g) was significantly higher than the median concentration collected in 2018 (2.7 ng/g) [9]. From a safety analysis perspective, there are many sources of safety risks for dairy products besides the common melamine. For example, sodium thiocyanate can be used as a preservative in milk, but it is also a goitrogen. At high concentrations, it can inhibit iodine and tyrosine oxidation in the human body, which may induce goiter. It has the most significant impact on pregnant women, children, and iodine-deficient patients. Currently, it is banned in many countries [10]. Zhang et al. developed an identification method based on surface-enhanced Raman spectroscopy (SERS) with a detection limit of 5 × 10^−7^ g/mL [11]. Considering the residue and safety risks of carbon-based materials in milk, Nunes et al. developed a multi-walled carbon nanotube detection method based on Fourier transform Raman spectroscopy and partial least squares (PLS), with a detection capability of up to 0.1 µg/mL [12]. Huang et al. used Raman spectroscopy combined with PLS regression and backpropagation artificial neural network (BP-ANN) to predict and analyze milk acidity, which can be used to evaluate milk freshness and identify acidity neutralization caused by the illegal addition of Na_2_CO_3_ [13]. Similar indicator detection methods provide rich technical support for the risk management of dairy products [14].

Quality analysis is a further development of safety assessment, which strengthens the quality consistency of dairy products on the basis of ensuring safety. The concept of quality can be understood as inversely proportional to variability [15]. When significant changes in quality characteristics are detected, quality mutations may occur, such as differences between dairy products of different brands and species. As reported by the media, for example, in 2016, there was an incident in Shanghai where some dairy products were counterfeited, with some low-priced dairy products being labeled as high-priced products by criminals in order to seek price differences, but these products themselves are qualified [16]. In addition, for dairy products from different species sources, such as camel milk, donkey milk, mare milk, goat milk, and cow milk, if the labeling does not match, it may lead to serious health risks, especially for individuals who are allergic to adulterated ingredients [17,18,19]. Ostovar Pour et al. used spatially offset Raman spectroscopy combined with chemometric methods to effectively distinguish eight different types of cheese [20]. Ullah et al. developed a method for distinguishing cow and buffalo milk based on the combination of Raman spectroscopy and chemometric techniques. They also found that the Raman peaks at 1150 and 1510 cm^−1^ corresponding to β-carotene are important biomarkers, as these two peaks only exist in cow milk and not in buffalo milk [21].

Raman spectroscopy, as an important chemical characterization technique for dairy products, can obtain relevant molecular vibration information. Compared with common chromatographic and mass spectrometry characterization techniques, its signal acquisition does not require pre-treatment and is relatively fast. Generally speaking, chromatographic operations require several hours of sample pre-treatment time (such as solvent extraction), followed by several minutes to tens of minutes for each sample detection. Mass spectrometry operations also often require several hours of sample pre-treatment, followed by several seconds to minutes for each sample detection time. Raman spectroscopy can be tested without pre-treatment, and the detection time for each sample is only a few seconds to a few minutes. The detection time for multidimensional Raman spectroscopy is slightly longer, which is also related to different perturbation designs. Compared with infrared spectroscopy, Raman spectroscopy can directly test water-containing samples. In addition, Raman spectroscopy can be portable, dairy products can be tested without pre-treatment, and non-invasive testing can be conducted to study dairy products through non-absorbent packaging [22]. Especially in recent years, the efficient combination of Raman spectroscopy and machine learning algorithms has brought about rapid development in the spectral characterization and intelligent analysis of dairy products [23]. Khan et al. investigated the online measurement of raw milk components based on Raman spectroscopy and found that the combination of spectroscopy and the PLS regression model can effectively predict the content of fat, protein, and lactose, which has the potential for online monitoring of dairy quality [24]. Genis et al. used Raman spectroscopy to investigate and determine the sources of fats in margarine, corn, and palm oil present in white and ultra-filtration cheese samples. The origins and adulteration ratios of these foreign fats/oils were determined using partial least squares discriminant analysis (PLS-DA) and PLS evaluation [25].

In this review, we will focus on the research on quantitative identification of dairy products based on Raman spectroscopy in recent years, with a particular emphasis on dairy product discrimination based on the full spectrum or feature spectrum of Raman spectroscopy, as well as dairy product identification research based on similarity measurement methods or machine learning algorithms, in order to further expand the research summary rules for reference in related fields and propose future challenges.

## 2. Characterization of Dairy Products Based on Multidimensional Raman Spectroscopy

**Figure 1:**
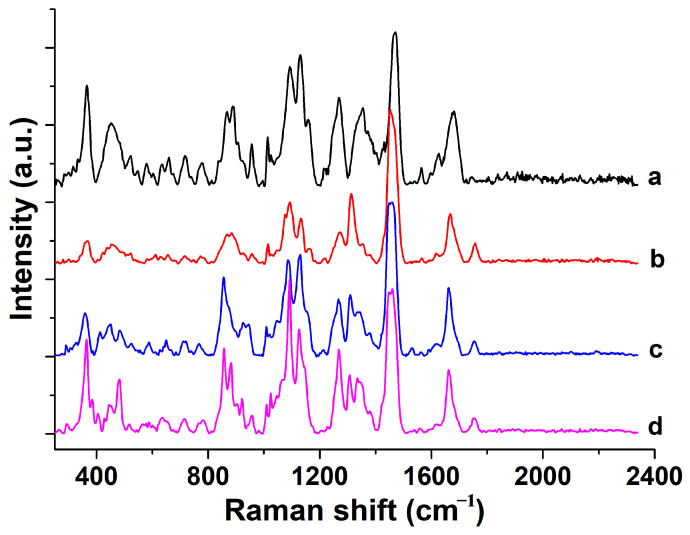
Raman spectra of Nestle skim cow milk powder (**a**), Nestle whole cow milk powder (**b**), Meisu Jia’er infant milk powder (**c**), and Jiabeiaite infant goat milk powder (**d**) [30].

There have been multiple reports on the characterization of dairy products based on Raman spectroscopy, which helps us understand the relevant Raman spectral characteristics of dairy products. Almeida et al. used Fourier transform Raman spectroscopy to characterize whole milk, low-fat milk, and skim milk powder and further combined principal component analysis (PCA) and PLS-DA techniques to perform multivariate analysis and classification of milk powder samples adulterated with whey [26]. From the Raman spectrum, it can be clearly seen that whole milk powder has a vibration peak at 1746 cm^−1^, while low-fat milk powder has a weaker peak, and skim milk powder does not have this peak. Ferreira et al. used a handheld Raman spectrometer to analyze the signals of goat milk and cow milk powder. The authors found a peak at 1154 cm^−1^, which is characteristic of carotenoids and does not exist in pure goat milk samples. Subsequently, the authors used spectral preprocessing combined with data-driven soft independent modeling of class anatomy (DD-SIMCA) to achieve sample classification [27]. There are also some dairy products whose Raman spectra display information about additive ingredients, such as aspartame, often added as a sweetener in yogurt, which enhances the peak originating from protein phenylalanine at 1014 cm^−1^ in the Raman spectrum, and a new ring vibration peak appears at 1614 cm^−1^ [28]. Reiner et al. used Raman spectroscopy for online process control in milk production and found that seven Raman peaks were related to fat molecules, and the Raman peak at 355 cm^−1^ was related to lactose [29]. Figure 1 shows the Raman spectra of different types of dairy products collected by our research group [30]. Raman spectroscopy was performed using a portable Raman spectrometer, model ProTT-EZRaman-D3, manufactured by Enwave Optronics in the United States. The detection conditions were a laser wavelength of 785 nm, a laser power of 450 mW, a charge-coupled device temperature of −85 °C, a laser exposure time of 50 s, a spectral resolution of 1 cm^−1^, and a spectral range of 250–2339 cm^−1^. The detection process involved adding each dairy powder sample into an independent small hole of a 96-well plate and then directly irradiating the sample with a Raman spectroscopy probe to collect Raman signals. Four different brands of dairy product samples were purchased from Suguo Supermarket in Nanjing, China, for the experiment, namely, Nestle skim cow milk powder (a), Nestle whole cow milk powder (b), Meisu Jia’er infant milk powder (c), and Jiabeiaite infant goat milk powder (d). The corresponding main spectral peak labeling information can be found in Figures S1–S4, and the corresponding substance content information of each brand sample is shown in Table S1. It can be seen that Raman spectra can well reflect the composition information of substances such as fat, carbohydrates, and proteins in dairy products. Combined with the existing literature reports on dairy products [24,26,30,31], the vibration and substance assignments of the main Raman spectral peaks were carried out, as shown in Table 1. Brands a and b are, respectively, skimmed milk powder and whole milk powder from the same manufacturer. The figure shows that the Raman peaks related to fat in skimmed milk powder are missing at 1757 cm^−1^ and 1311 cm^−1^, while the Raman peak signal amplitudes related to carbohydrates and proteins in skimmed milk powder appear more pronounced. Brand c is infant whole milk powder, which has significant differences in the position and shape of a small amount of Raman peaks compared to brand b. Brand d is infant goat milk powder, which is different from brands a, b, and c, which are all cow milk powder, and there are also some differences in Raman peaks.

**Figure 2:**
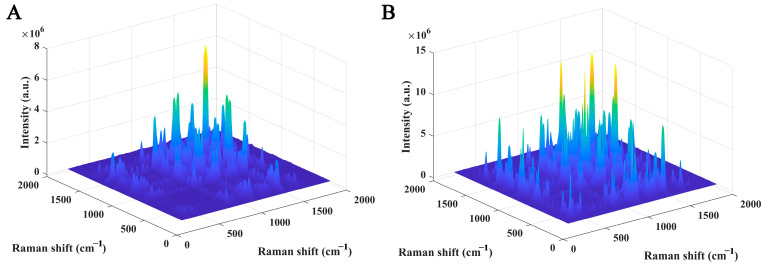
Two-dimensional correlation Raman spectra and three-dimensional images of cow milk (**A**) and goat milk powder (**B**) [30].

In recent years, Professor Noda has proposed a method for constructing high-dimensional spectra. The basic idea is to obtain a series of spectra by designing an external perturbation such as temperature, concentration, pH, reaction, and other physical and chemical effects and then reconstruct the high-dimensional spectra using two-dimensional correlation analysis, which has the advantage of improving spectral resolution [33]. The basic construction mechanism is to examine the changes in Raman spectral signal intensity y(ν,t) during the transition from the initial stage T_1_ to reaching the new equilibrium state T_2_ of the dairy system with an external disturbance variable of *t*. Collect Raman spectral signals from various time periods during the entire change process of the experimental system, use the average spectrum or specified spectrum of the entire system change process as the reference spectrum, and construct a two-dimensional correlated Raman spectrum based on the formula $X(\nu_{1},\nu_{2})=\Theta (\nu_{1},\nu_{2})+i\Psi (\nu_{1},\nu_{2})$ [34]. At present, this technology is mainly applied in the field of infrared spectroscopy, such as Lafi et al.’s construction of two-dimensional correlated Fourier transform infrared spectroscopy, which achieves quantitative detection of carbohydrate adulteration such as sucrose, lactose, and starch in milk powder [35]. Huang et al. combined two-dimensional infrared correlation spectroscopy with N-way partial least squares discriminant analysis (NPLS-DA) to achieve accurate discrimination of adulterated milk. The results showed that the new method had a prediction set discrimination accuracy of 100%, while the discrimination accuracy using traditional three-dimensional (3D) stacked graphs was 77.8% [36]. Zhang et al. constructed two-dimensional correlated Raman spectroscopy with laser integration time as the external perturbation, characterized different brands of fresh milk samples, and combined feature extraction and Euclidean distance measurement to achieve fresh milk brand discrimination [37]. Gu et al. also characterized the Raman spectra of cow milk and goat milk powder and found that goat milk powder had spectral peaks at 882 cm^−1^ and 385 cm^−1^, while cow milk powder had no obvious spectral peaks at these two locations. Subsequently, the authors constructed a two-dimensional Raman spectroscopy analysis of the milk powder, characterized it from a high-dimensional space, and combined it with the Euclidean distance algorithm to achieve a sample classification [30]. The two-dimensional Raman spectral peaks of dairy products are shown in Figure 2, indicating that more spectral details are presented from the high-dimensional space. Intuitively, it can be clearly seen that there are significant differences in the spectra between the goat milk powder and cow milk powder collected in the experiment.

## 3. Standardization and Preprocessing of Raman Spectroscopy for Dairy Products

**Figure 3:**
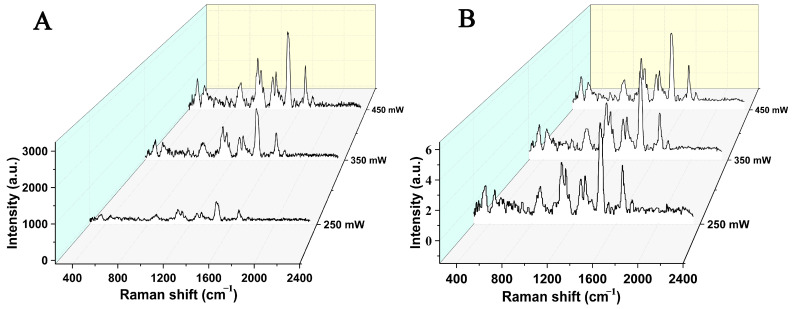
Original Raman spectra (**A**) and Raman spectra after mean normalization and wavelet denoising (**B**) of milk powder under different laser power conditions [39].

From the aforementioned literature analysis, it can also be seen that there are certain differences in the spectral characterization results of different research groups, which may be due to differences in sample sources or instrument equipment. Therefore, this suggests that it is necessary to conduct standardization research on data to improve the transferability of spectral data [38]. Zhang et al. investigated the characterization of milk powder Raman spectra under different laser irradiation intensity settings and, combined with mean normalization and wavelet denoising processing, effectively improved the consistency of spectral characterization [39]. As shown in Figure 3, it can be seen that for Raman spectroscopy data of the same brand of dairy products collected under different laser intensity conditions, after standardization and wavelet denoising processing, the consistency of the spectra can be significantly enhanced, effectively reducing the interference of noise. Figure 3A shows the Raman spectroscopy data of dairy products collected under different laser intensities, namely 250 mW, 350 mW, and 450 mW. Figure 3B shows the data obtained after mean normalization (formula as follows) and db1 wavelet denoising.

$$\mathrm{Mean}\;\mathrm{normalization}\;\mathrm{formula}:\;y_{i}=\frac{x_{i}-\bar{x}}{s}$$

In the formula, $x_{i}$ represents the intensity value of the original Raman spectrum, $y_{i}$ represents the intensity value of the Raman spectrum after mean normalization; $\bar{x}$ represents the average value of $x_{i}$, and the calculation formula is $\bar{x}=\frac{\sum_{i=1}^{n}x_{i}}{n}$; s represents the standard deviation, and the calculation formula is $s=\sqrt{\frac{\sum_{i=1}^{n}{\left(x_{i}-\bar{x}\right)}^{2}}{n-1}}$; and n represents the number of shifts in a Raman spectrum.

**Figure 4:**
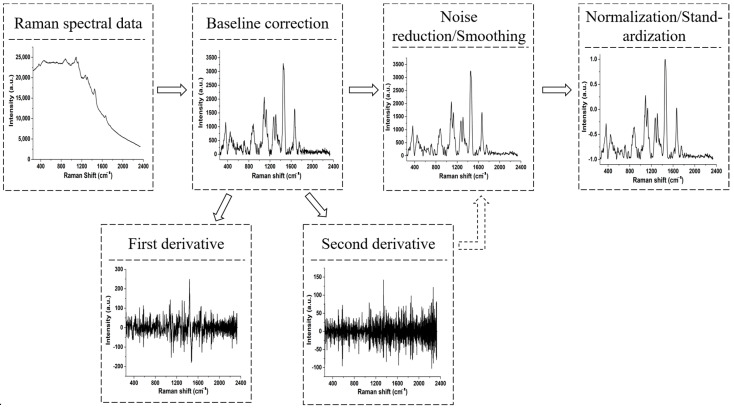
Schematic diagram of preprocessing process for Raman spectroscopy.

There are many preprocessing methods for Raman spectroscopy, and when conducting quantitative-related research, it is often necessary to choose appropriate preprocessing techniques based on specific algorithms [40]. Tian et al. studied the adulteration of raw milk based on Raman spectroscopy, PLS-DA, and PLS regression. They found that after preprocessing with the first derivative and variable selection optimization with variable importance in the projection, a model with excellent performance can be obtained [41]. Zhao et al. built a brand discrimination of infant rice noodles based on the Raman spectrum and extreme learning machine (ELM). The research found that the recognition rate was significantly improved after coif3 wavelet denoising and 0~0.1 normalization processing [42]. Karachaglar et al. developed a discrimination method based on Raman spectroscopy preprocessing, including the first derivative, Savitzkye–Golay (filter width: 15; polynomial order: 2), and mean centering operation, as well as PCA. This method can be employed for the identification of non-milk-based fats or oils in milk cream and yogurt [43]. Based on the above literature analysis and combined with our group’s work experience, a recommended process for Raman spectroscopy preprocessing of dairy products can be summarized, as shown in Figure 4. Firstly, the measured data are collected using a Raman spectrometer. After baseline correction, wavelet denoising or Savitzkye–Golay smoothing can be used to remove noise interference, and then the influence of dimensionality can be removed through standardization or normalization. Sometimes, research groups may use derivative processing, such as the first and second derivatives, to optimize data. However, based on our previous research findings, the improvement effect of derivative processing on recognition algorithms is often limited [44]. It is recommended to carefully choose derivative processing when combining algorithms in actual Raman spectroscopy preprocessing.

## 4. Quantitative Recognition of Dairy Products Based on Feature Extraction

When combining Raman spectroscopy with quantitative analysis methods for dairy product evaluation, research has found that using the collected full spectrum directly does not always yield the best computational results [45]. In order to improve the analysis efficiency, researchers have developed various spectral feature extraction methods. Dewantier et al. conducted a classification analysis of the maturity level and manufacturer of cheddar cheese using Raman and infrared spectroscopy combined with PCA. PCA is a common data conversion method that can achieve dimensionality reduction and feature extraction in spectral data. The article also explains the spectral bands and component attribution of the extracted principal components [46]. Ni et al. compared four spectral feature extraction algorithms, namely the successive projections algorithm (SPA), uninformative variable elimination (UVE), random frog (RF), and competitive adaptive reweighted sampling (CARS), and found that CARS had the best performance in assisting milk adulteration with support vector machine (SVM) classifier discrimination [47].

Another group of scholars, considering the complexity and poor interpretability of mathematical transformation methods, has attempted to explore various direct extraction methods for spectral features by combining spectral chemical information. For example, Zhang et al. conducted chemical feature extraction on dairy products based on their Raman spectral peak intensity, peak area, and peak ratio. They successfully classified the experimental samples by combining the Euclidean distance and quality fluctuation control charts. The results revealed that the discriminative ability of these three feature extraction methods increased in sequence, and the chemical information attribution was clear [48]. Zhang et al. used the SVM algorithm for discriminant analysis of different brands of pasteurized milk. When combined with appropriate spectral feature intervals and normalization processing, the recognition rate increased from 58% to 93%, and the computation time was shortened by about 75%. The accuracy and efficiency were greatly improved, demonstrating the importance of spectral feature extraction [44].

Table 2 presents the relevant Raman spectral feature selection methods reported in recent years, and it can be seen that the Raman spectral feature extraction methods can be roughly divided into two categories. One type is the data conversion type, such as PCA. The other type is the spectral feature direct screening type. Both methods have their own characteristics. The mathematical transformation method has fine feature extraction, but the operation is relatively complex, and the interpretability is relatively poor. The direct screening method has good interpretability and is a relatively simple operation. However, the efficiency of feature extraction is related to the actual spectral resolution obtained, and sometimes feature screening is difficult.

## 5. Quantitative Identification of Dairy Products Based on Similarity Evaluation

**Figure 5:**
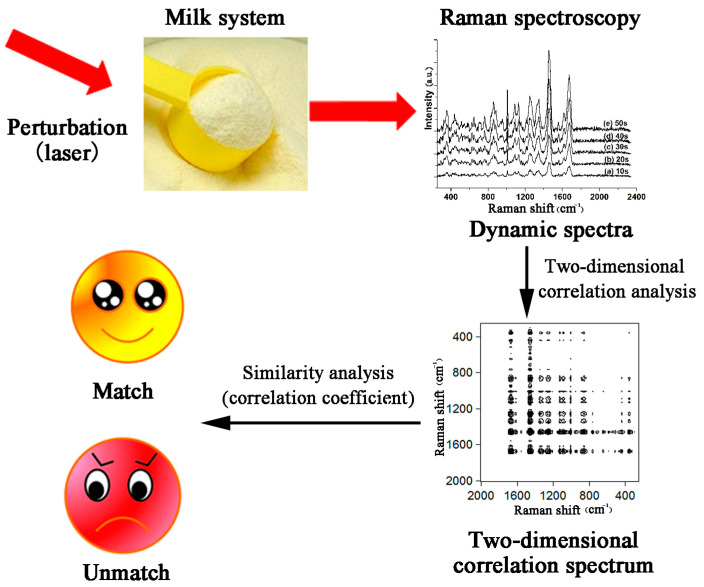
Discriminant analysis of dairy products based on similarity measurement [54].

After obtaining Raman spectroscopy data of dairy product samples, qualitative identification can be performed with the naked eye, followed by quantitative evaluation using similarity assessment methods [52]. There are two commonly used similarity measurement methods. One is represented by the correlation coefficient, cosine of angle, etc. The larger the similarity between the sample spectra, the closer the correlation coefficient value is to one. Conversely, the smaller the similarity between the sample spectra, the closer the correlation coefficient value is to zero. Arroyo Cerezo et al. obtained spatially offset Raman spectra of sliced cheese from different species, including cows, sheep, goats, etc., and conducted nearness index (NEAR) similarity index analysis. They found that cheese made from milk of the same animal species had a high similarity (NEAR > 0.8), higher than cheese produced from milk of different animal species [53]. Zhang et al. used a laser as a perturbation to obtain two-dimensional Raman spectra of various milk powder products. Due to the prominent differences in sample details in high-dimensional spectra, people can intuitively distinguish between different brands of samples. Subsequently, the authors used the correlation coefficient method to quantitatively analyze the differences between each experimental sample. The results showed that the correlation coefficient between samples of the same brand and their mean value reached 0.991 ± 0.003, while the correlation coefficient between samples of other brands and the mean value of the experimental group was only 0.596~0.761, which effectively achieved brand discrimination of dairy products [54]. The research schematic is shown in Figure 5.

Another measurement method is represented by the Euclidean distance, Mahalanobis distance, etc. The greater the similarity between sample spectra, the smaller the Euclidean distance value. Conversely, the greater the difference between sample spectra, the larger the Euclidean distance value. Zhang et al. constructed two-dimensional correlated Raman spectra of two different brands of fresh milk and found significant differences in the Raman spectral ratio of 1015/1455. With the help of a Euclidean distance quantification calculation, differentiated quantification and graphical display were achieved [37]. In addition, after obtaining the similarity measure of dairy products, it is also possible to consider combining the quality control chart method to further draw a quality fluctuation control chart for the research object and combining the control chart judgment rules to determine the quality stability [48]. Zhang et al. developed a yogurt brand identification method based on the k-nearest neighbor algorithm and found that using a nearest neighbor algorithm constructed based on the Mahalanobis distance can achieve better recognition accuracy compared to the Euclidean distance [55].

## 6. Quantitative Identification of Dairy Products Based on Machine Learning Algorithms

When the quantity of dairy products is sufficient, and the sample detection data have accumulated to a certain extent, the differences between samples can be intelligently distinguished through machine learning algorithms. The quantitative analysis of Raman spectra of dairy products based on machine learning has received widespread attention and developed rapidly in recent years and can be mainly divided into two types: classification and prediction. The basic idea is to first perform relevant model calculations based on known Raman spectroscopy data and then use this model to predict the classification or composition regression of unknown samples [56,57,58]. In practical cases of dairy product identification, it is often necessary to combine specific algorithms for spectral parameter processing and optimization. Ni et al. reported a screening method for identifying adulterated ultra-high-temperature sterilized milk in pasteurized milk and proposed a discrimination model combining lactose index screening, feature extraction, and the SVM algorithm to achieve an accurate identification of experimental samples [47]. Amjad et al. achieved the classification of experimental dairy samples by combining PCA dimensionality reduction with the random forest algorithm on Raman spectra of milk samples from different species, including cows, buffaloes, goats, and humans [59]. Zhang et al. investigated the combination of Raman spectroscopy and the random forest algorithm to achieve rapid discrimination of different brands of soybean milk powder. After optimization, they found that the recognition effect was best under the conditions of db2 wavelet, normalization, principal component analysis, and 30 decision trees [60]. Li et al. collected Raman spectra of goat milk and cow milk and used PCA to classify these samples. Then, combined with PLS regression, quantitative prediction of adulterated cow milk in goat milk was achieved [61]. Yazgan et al. used Raman spectroscopy combined with PLS-DA to identify the types of cow, goat, ewe, and mixed adulterated milk in raw milk and pasteurized milk [62]. Zhang et al. studied the Raman spectroscopic characterization of cheese samples under different laser integration intensities and combined wavelet denoising, normalization, and PCA processing to effectively improve the recognition accuracy of the ELM algorithm and optimize the characterization parameters suitable for the discrimination model [63]. Yaman et al. studied the combination of Raman spectroscopy and infrared spectroscopy with PLS regression and the soft independent modeling of class analogy (SIMCA) for the identification of cow milk adulteration in goat milk. Proposing β-carotene as a biomarker, combined with the spectral band feature, is expected to predict β-carotene content and distinguish adulterated mixtures [64].

Meanwhile, with the continuous emergence of new deep learning algorithms, such as convolutional neural networks, they have new characteristics in feature extraction and other performance aspects [65]. Li et al. compared the brand discrimination of dairy products using Raman spectroscopy combined with SVM, ELM, and convolutional neural network (CNN) algorithms. The results showed that the recognition rates of the three algorithms, when directly combined with the full spectrum, were 33.3%, 92.5%, and 100%, respectively. After normalization, the SVM recognition rate could be significantly improved. After combining with specific spectral ranges, the recognition rates of each algorithm could reach 100%, indicating that there are differences in the applicability conditions of each machine learning algorithm. CNNs achieve optimal performance in processing raw data, which is closely related to their excellent feature extraction performance [51].

**Figure 6:**
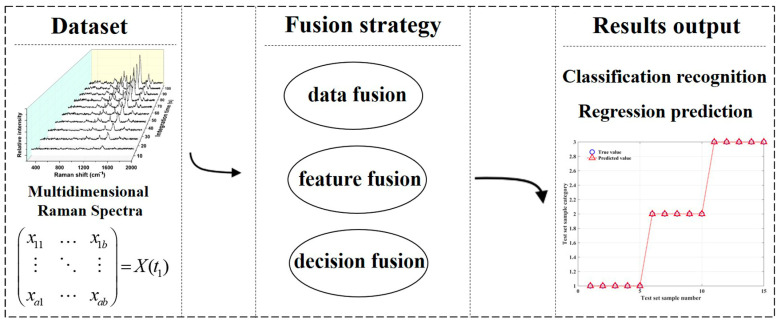
Schematic diagram of spectral data fusion research.

Recently, in order to overcome the limitations of single-modal representation or single-algorithm operation, fusion research strategies have attracted more and more scholars’ attention. Mohammadi et al. first characterized seven types of Irish milk using near-infrared, mid-infrared, and Raman spectra. Then, single-spectrum data, fused-spectrum data, PLS-DA, and sequential and orthogonalized partial least squares linear discriminant analysis (SO-PLS-LDA) were sequentially combined for milk classification. The results showed that the Raman spectrum recognition rate was the highest when a single spectrum was combined with PLS-DA, but only 85.71%, and the recognition rate when three spectra were fused with PLS-DA was only 85.71%. After the fusion of three spectra and the combination with SO-PLS-LDA, the recognition rate can reach 95%, and the recognition effect is significantly improved [66]. Wang et al. successfully constructed an SVM regression based on intermediate data fusion to predict the optimal storage time for the Vis NIR and Raman spectra obtained from infant formula milk powder [67]. Feng et al. investigated the use of a single light gradient boosting machine (Light GBM), SVM, random forest and extreme gradient boosting (XGBoost) algorithms, as well as fusion algorithms for dairy product brand classification and fat content prediction. The results showed that the classification accuracy of a single algorithm was around 90%, while the fusion algorithm could reach 99%, and the fat content prediction also performed better [68]. From the analysis of the existing literature, it can be concluded that the fusion analysis based on Raman spectroscopy can be roughly classified into three strategies: data layer fusion, feature layer fusion, and decision layer fusion, each with its own characteristics, as shown in Figure 6. Data layer fusion is simple and easy to implement, but it may also introduce redundant data or noise into the model to affect recognition performance. Feature layer fusion is closely related to feature extraction methods, and generally, only a small number of features can replace the original multidimensional data, which is expected to improve recognition performance and computational efficiency. The fusion of multiple algorithm advantages at the decision-making level is beneficial for improving model performance, but the computational complexity is relatively high.

## 7. Conclusions and Prospects

Quantitative techniques based on Raman spectroscopy play a crucial role in addressing quality and safety risks in dairy products. This work first summarizes and analyzes the characteristics of dairy product characterization data based on multidimensional Raman spectroscopy, revealing that there are certain differences in the spectra of dairy products of different species and brands. Then, the standardization, preprocessing, and feature extraction methods of Raman spectra are summarized in sequence, revealing that there are multiple options for normalization, wavelet denoising, and feature extraction methods. Appropriate processing can improve the display of spectral signals and enhance the classification and prediction effects of subsequent models. Finally, quantitative analysis methods are developed from two aspects: similarity measurement and machine learning algorithms, revealing that similarity evaluation can quantify the differences between sample spectra, which is beneficial for small batch sample discriminant analysis. At the same time, machine learning algorithms are suitable for identifying and analyzing a large number of samples, and there are matching requirements between related algorithms and spectral parameters and processing, presenting a trend of integrated development of the data layer, feature layer, and decision layer.

In terms of future trend analysis, at the level of Raman spectroscopy data for dairy products, standardized multidimensional Raman spectroscopy collection methods and spectral databases for different dairy species and brands have not yet been formed. The construction methods and cases of high-dimensional spectra are still relatively limited, and continuous research is still needed to provide more research solutions. In addition, with the iterative development of deep learning algorithms, new algorithms that integrate feature extraction and other functions have rapidly emerged. However, the problem of the model black box still exists, and the problem of model interpretability will become a new challenge.

## Figures and Tables

**Table 1:** The main peak assignments of the Raman spectra of the dairy products [24,26,30,31,32].

Raman Shift (cm^−1^) Brand a	Raman Shift (cm^−1^) Brand b	Raman Shift (cm^−1^) Brand c	Raman Shift (cm^−1^) Brand d	Assignment	Possible Component Attribution
-	1757	1753	1754	ν (C=O) ester	Fat
1678	1667	1661	1662	ν (C=O) amide I	Protein
1625	1618	1616	1617	ν (C=C) ring	Protein
1565	1565	1562	1558	δ (N–H), ν (C–N) amide II	Protein
1471	1453	1457	1457	δ (CH_2_)	Fat, Carbohydrate
1355	1353	1338	1335	δ (C–H), ν (C–O)	Carbohydrate
-	1311	1309	1307	τ (CH_2_)	Fat
1270	1272	1267	1268	γ (CH_2_)	Carbohydrate
1130	1131	1130	1125	ν (C–O) + ν (C–C) + δ (C–O–H)	Carbohydrate
1091	1092	1087	1093	ν (C–O) + ν (C–C) + δ (C–O–H)	Carbohydrate
1014	1013	1008	1008	ν (C=C) ring	Protein
955	953	946	957	δ (C–O–C) + δ (C–O–H) + ν (C–O)	Carbohydrate
925	925	925	921	δ (C–O–C) + δ (C–O–H) + ν (C–O)	Carbohydrate
889	884	-	882	δ (C–C–H) + δ (C–O–C)	Carbohydrate
866	869	855	857	δ (C–C–H) + δ (C–O–C)	Carbohydrate
778	772	767	784	δ (C–C–O)	Carbohydrate
715	715	713	711	ν (C–S)	Protein
659	658	650	-	δ (C–C–O)	Carbohydrate
634	631	628	639	δ (C–C–O)	Carbohydrate
580	580	588	585	δ (C–C–O) + τ (C–O)	Carbohydrate
520	523	525	518	Glucose	Carbohydrate
-	-	482	481	δ (C–C–C) + τ (C–O)	Carbohydrate
452	453	448	445	δ (C–C–C) + τ (C–O)	Carbohydrate
-	-	409	405	Lactose	Carbohydrate
-	-	-	385	Lactose	Carbohydrate
364	369	358	365	Lactose	Carbohydrate
-	-	290	294	Lactose	Carbohydrate

Note 1. Brand a: Nestle skimmed cow milk powder; brand b: Nestle whole cow milk powder; brand c: Meisu Jia’er infant cow milk powder; and brand d: Jiabeiaite infant goat milk powder. Note 2. ν: stretching vibration; δ: deformation vibration; τ: twisting vibration; and γ: out-of-plane bending vibration.

**Table 2:** List of Raman Spectral Feature Extraction Methods.

Feature Extraction Method	Application Situation	References
PCA	Classification of male and female buffalo milk samples	[49]
PCA	Quantitative determination of whey in raw milk	[50]
SPA, UVE, RF, and CARS	Identification of adulteration between pasteurized milk and ultra-high temperature sterilized milk	[47]
Raman peak height, peak area, and peak ratio	Quality control of dairy products from different brands	[48]
Moving window selection, spectral band selection, and fusion	Identification of different brands of pasteurized milk	[44]
Spectral intervals extraction and fusion	Quality discrimination of different brands of dairy products	[51]
High-dimensional Raman peak ratio	Identification of fresh milk samples	[37]

Note 1. PCA: principal component analysis; SPA: successive projections algorithm; UVE: uninformative variable elimination; RF: random frog; and CARS: competitive adaptive reweighted sampling.

## Data Availability

Dataset available on request from the authors.

## Supplementary Materials

The following supporting information can be downloaded at: https://www.mdpi.com/article/10.3390/molecules30020239/s1, Figure S1: Raman spectra of Nestle skim cow milk powder; Figure S2: Raman spectra of Nestle whole cow milk powder; Figure S3: Raman spectra of Meisu Jia’er infant milk powder; Figure S4: Raman spectra of Jiabeiaite infant goat milk powder; Table S1: The main nutritional content of different brands of dairy products.
