# Supplementary material for: Technology for the Quantitative Identification of Dairy Products Based on Raman Spectroscopy, Chemometrics, and Machine Learning
Source: Molecules. 2025 Jan 9;30(2):239. doi: 10.3390/molecules30020239 (PMC11767359; doi:10.3390/molecules30020239)
Supplement: Supplementary file 1 [file molecules-30-00239-s001.zip › molecules-3349596-supplementary.pdf]

Quantitative Identification Technology of Dairy Products Based on Raman Spectroscopy, Chemometrics, and Machine Learning

Zheng-Yong Zhang [1], Jian-Sheng Su [1] and Huan-Ming Xiong [2,*]

1 School of Management Science and Engineering, Nanjing University of Finance and Economics, Nanjing 210023, Jiangsu, China
2 Department of Chemistry and Shanghai Key Laboratory of Molecular Catalysis and Innovative Materials, Fudan University, Shanghai 200438, China
* Correspondence: hmxiong@fudan.edu.cn

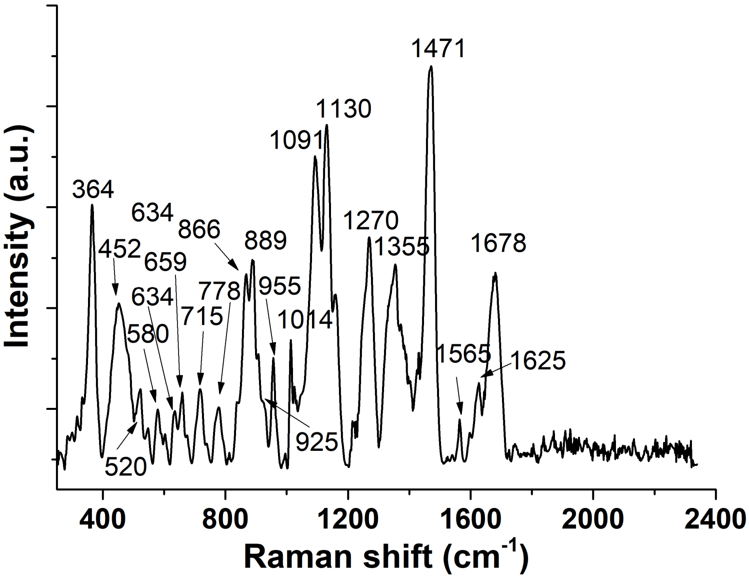

Figure S1. Raman spectra of Nestle skim cow milk powder.

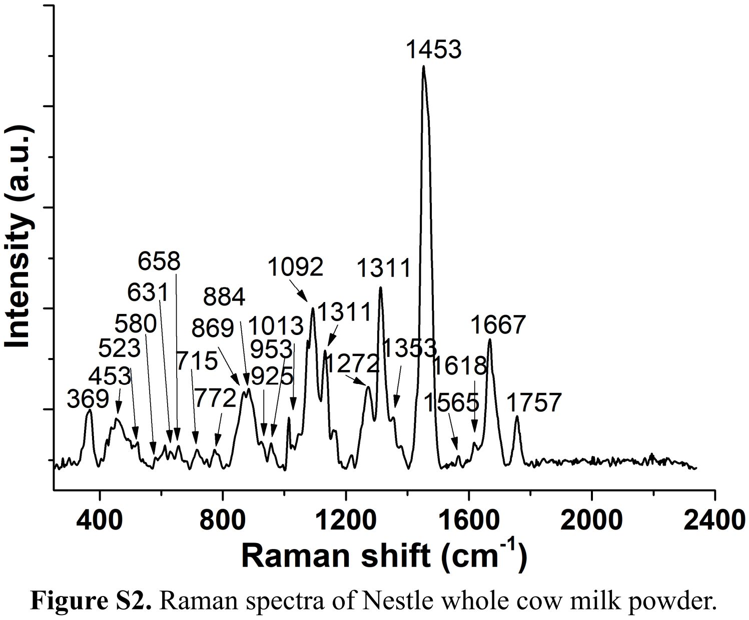

Figure S2. Raman spectra of Nestle whole cow milk powder.

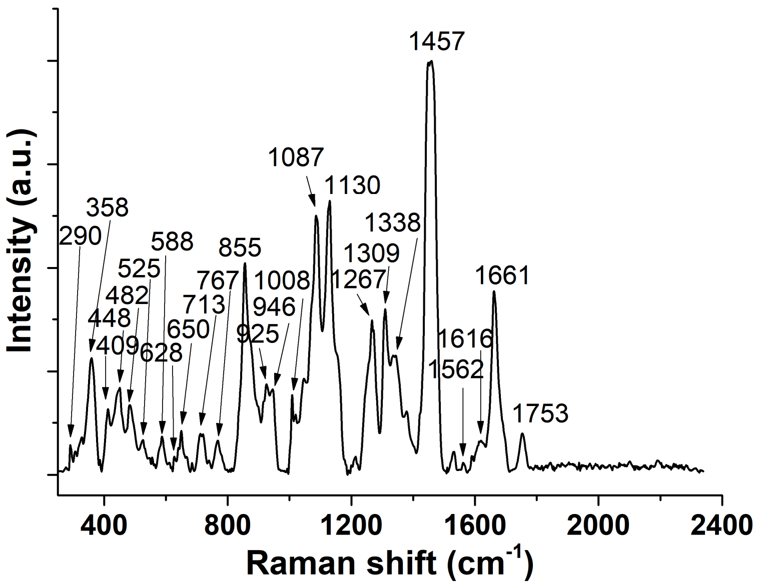

Figure S3. Raman spectra of Meisu Jia'er infant milk powder.

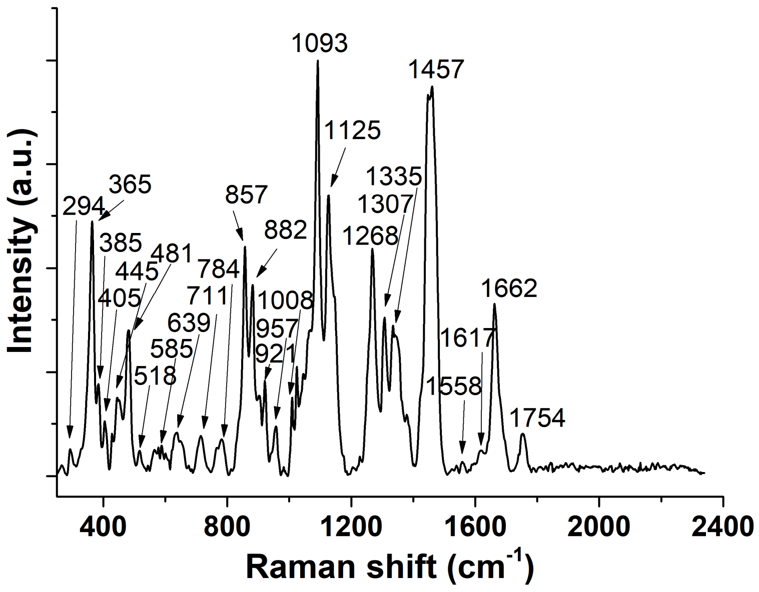

Figure S4. Raman spectra of Jiabeiaite infant goat milk powder.

Table S1. The main nutritional content of different brands of dairy products.

	fat	carbohydrates	proteins
Nestle skim cow milk powder	1.5g/100g	53g/100g	33g/100g
Nestle whole cow milk powder	27g/100g	35g/100g	24g/100g
Meisu Jia'er infant milk powder	22.5g/100g	51.2g/100g	16.6g/100g
Jiabeiaite infant goat milk powder	19.7g/100g	60g/100g	10.8g/100g
